# Temporal Changes in Individualism and Their Ramification in Japan: Rising Individualism and Conflicts with Persisting Collectivism

**DOI:** 10.3389/fpsyg.2017.00695

**Published:** 2017-05-23

**Authors:** Yuji Ogihara

**Affiliations:** ^1^Department of Cognitive Psychology in Education, Graduate School of Education, Kyoto UniversityKyoto, Japan; ^2^Department of Psychology, University of California, Los AngelesLos Angeles, CA, USA

**Keywords:** individualism, cultural change, temporal change, Japan, socioeconomic environment, name, cultural product, uniqueness

## Abstract

Many studies have shown that American culture has become more individualistic over time. However, it was unclear whether other cultures, especially East Asian cultures, have also shifted toward greater individualism. Therefore, this article reviewed studies investigating temporal changes in individualism in Japan and their ramifications on psychology and behavior. Japan has experienced rapid and dramatic economic growth and urbanization and has adopted more social systems based on individualistic concepts in various contexts (e.g., workplace, school). Recent studies have suggested that, along with these socioeconomic changes, Japanese culture has become more individualistic over time. Specifically, the divorce rate increased and household size decreased. Moreover, people give more unique names to their children and dogs, and individualistic words such as “individual” and “uniqueness” appear more frequently in newspapers. Furthermore, social values became more individualistic. Yet, it has also been shown that some collectivistic values still remain. As a result, people have difficulty in adapting to this coexistence, which injures interpersonal relationships and well-being. This paper discussed how Japanese culture changed over time and how such changes affected Japanese psychology and behavior.

The main purpose of this paper is to overview research investigating cultural changes in **individualism** and their ramifications in Japan. This paper consists of six sections. The first section offers a brief explanation of the theoretical/practical background of this paper and its implications. I will explain how research examining cultural changes is important by presenting some possible contributions it can make to both academia and society at large.

KEY CONCEPT 1 **Individualism**

Individualism is defined as “a social pattern that consists of loosely linked individuals who view themselves as independent of collectives; are primarily motivated by their own preferences, needs, rights, and the contracts they have established with others; give priority to their personal goals over the goals of others; and emphasize rational analyses of the advantages and disadvantages to associating with others” (Triandis, 1995, p. 2). Importantly, “[t]he core element of individualism is the assumption that individuals are independent of one another” (Oyserman et al., 2002, p. 4).

The second section summarizes the accumulated findings regarding temporal changes in individualism in American culture. The amount of previous literature examining temporal changes in American culture is relatively large compared to work looking at temporal changes in other cultures. Due to space limitation, I briefly introduce studies that are relevant to studies in Japan.

The third section, which constitutes the core of this paper, reviews studies examining temporal changes in individualism in Japan. Compared to the well accumulated literature investigating cultural changes in individualism in the U.S., the amount of research examining cultural changes in Japan is relatively small. Culture gradually changes by interacting with its historical background, so the patterns of temporal change may differ across cultures (Inglehart and Baker, 2000). Thus, to reveal how culture in general changes over time and how culture and people make each other up, it is important to examine temporal changes in culture other than America. As cultural psychology has shown empirically that there are many cultural differences in psychology and behavior, there may be important cultural differences in temporal change as well.

The fourth section addresses the psychological ramifications of such changes in Japan. I will review how shifts in individualism are related to interpersonal relationships and well-being/happiness in a culture that has not yet become fully individualistic.

The fifth section summarizes this paper, and the sixth section raises some future directions for the investigation of cultural change.

## 1. The importance of researching cultural change

It is important to investigate cultural changes for at least four reasons. First, examining cultural changes contributes to a better understanding of the dynamic aspects of culture. Although some aspects of culture persist without change, we often realize that a culture in the present is not the same as it was 50 years ago. Cultural psychology emphasizes the mutual construction of culture and mind (Shweder, 1990), meaning that culture and mind make each other up. This posits dynamic and changeable processes. However, much research has compared cultures only at a single point in time and has not sufficiently examined these dynamic aspects of culture. By investigating how cultures change (or persist) over time, we can better understand the dynamic aspects of culture.

Second, investigating how and why cultures change over time can reveal how psychology and behavior are affected by social, economic, and ecological factors. This has been conducted at the individual and area (e.g., nation, state) levels (for reviews, see Oishi and Graham, 2010; Oishi, 2014). Temporal or historical analyses at the time (e.g., year, month) level can also find relationships between psychology/behavior and social/economic/ecological variables by focusing on variance at the group-level within a given timeframe (Ogihara, 2017a). This presents us richer information. Examples of these are given below (2-2; also see Ogihara, 2017a).

Third, revealing cultural changes may benefit researchers with regards to replication. Recently, researchers in psychology have paid more attention to the replicability of research results (e.g., Open Science Collaboration, 2015). One possible reason why research results cannot be replicated is cultural change. Psychological and behavioral tendencies can change depending on shifts in social, economic, and ecological environments. Thus, it is understandable that, due to changes in social, economic, and ecological environments, some findings obtained in the past are no longer found in the present. For example, prior research has shown that conformity, as measured with an Asch-type line judgment task (e.g., Asch, 1952), declined in the U.S. between the 1950s and 1990s (Bond and Smith, 1996). While it is one of the most famous and influential research findings in social psychology, this phenomenon has become less prevalent over time, which may lead to recent failures in experimental replication. Therefore, understanding how cultures change over time can reveal one plausible reason for replication failures.

Fourth, societies at large can benefit from investigating cultural changes. Given that the speed of globalization has increased and its influence is huge, cultures may change more dramatically and rapidly than before. Psychology and behavior may also be strikingly transformed. To solve social issues and prevent them in advance, it is effective to grasp what happens in the present scientifically and empirically by revealing how psychology and behavior have changed over time. For instance, if we can demonstrate how loneliness in a given nation changes over time in certain subgroups (e.g., sex, age, region) and examine group similarities and differences, we can understand the possible underlying mechanisms and deal with them more effectively.

## 2. Temporal changes in individualism in the U.S

### 2.1. Empirical evidence indicating greater individualism

Studies examining cultural changes in individualism in the U.S. suggest that there has been a rise in individualism (for detailed reviews, see Twenge, 2015; Greenfield, 2016). Here, because of space constraints, I will shortly overview studies that are relevant to studies in Japan.

#### 2.1.1. Divorce rates

The rate of divorce is a behavioral measurement which reflects individualistic tendency. In individualistic societies, family structure tends to be looser and freer relative to that in collectivistic societies (e.g., Triandis, 1995; Georgas et al., 2001). Thus, divorce rate is more likely to be higher in individualistic societies. Indeed, the divorce rate was correlated with other indices of individualism (the index developed by Hofstede, 1980, 2001; Triandis's rating of individualism-collectivism) and variables related to individualism, such as the rate of pronoun drop (Kashima and Kashima, 1998) and pathogen prevalence (e.g., Fincher et al., 2008; Murray and Schaller, 2010) at the national level (e.g., Diener et al., 1995; Lester, 1995; Toth and Kemmelmeier, 2009; Hamamura, 2012). Thus, divorce rate has been frequently used as an indicator of individualism (e.g., Diener et al., 1995; Vandello and Cohen, 1999; Hamamura, 2012; Yamawaki, 2012; Grossmann and Varnum, 2015).

The divorce rate in the U.S. increased between 1900 and 2009 (Hamamura, 2012; Grossmann and Varnum, 2015). In 1900, only 7.5 out of 100 couples divorced, but in 2009, 51.0 out of 100 couples experienced divorce. This rise in the rate of divorce is indicative of an increase in individualism in the U.S.

#### 2.1.2. Household size

Household size is another behavioral indicator of individualism. As mentioned above, in individualistic societies, family relationships are more likely to be freer and looser, leading people to live separately and independently of other family members. Thus in individualistic societies, household size tends to be smaller than in collectivistic societies. In fact, household size is correlated with another index of individualism (the index developed by Hofstede, 1980, 2001) and variables related to individualism, such as the rate of pronoun drop (Kashima and Kashima, 1998) and pathogen prevalence (e.g., Fincher et al., 2008; Murray and Schaller, 2010) at the national level (e.g., Hamamura, 2012). Thus, this index has been used in previous research (e.g., Hamamura, 2012; Grossmann and Varnum, 2015).

Household size in the U.S. decreased between 1860 and 2012 (e.g., Hamamura, 2012; Grossmann and Varnum, 2015). In 1860, the average household consisted of 5.6 people, but in 2012 the average was 3.1 people. This shift in household size is indicative of an increase in individualism in the U.S.

#### 2.1.3. Human baby names

Giving uncommon names to human babies is a valid indicator of individualism. Seeking uniqueness is one domain of individualism (e.g., Kim and Markus, 1999; Oyserman et al., 2002; Kim and Sherman, 2008; Taras et al., 2014). Indeed, an empirical examination has confirmed that rates of top 10 most common names were negatively correlated with an index of individualism (Hofstede et al., 2010) at the national level (Varnum and Kitayama, 2010). For example, the rates of the top 10 common names are low in the U.S., Canada, and Australia, while they are relatively high in Spain, Hungary, and Austria. Research has also shown that the rates of common names are lower in states where people recently settled than in states where people settled in the past in the U.S. and Canada. Prior research has shown that voluntary settlement promotes individualism (for a review, see Kitayama et al., 2010). Thus, in other words, the relative prominence of unique names in more recently settled states in these two nations is indicative of higher levels of individualism.

By using this index of individualism, Twenge and her colleagues examined temporal changes in individualism in the U.S. (Twenge et al., 2010, 2016). They computed the rate of common names (the most popular name, and the top 10, 25, and 50 most common names) between 1880 and 2015 in the U.S. The American government has collected almost all newborn names and displayed the rankings of the top common names each year during that period. They found that the rates of common names decreased for both boys and girls over this 130 year period, suggesting a rise in individualism in the U.S.

#### 2.1.4. Words in books

The increase in individualism has also been found in another cultural product, books. Researchers have used a database of huge amounts of books published in several languages (Google Books Ngram Viewer) to examine temporal changes in individualism. Researchers examining temporal changes using books have focused on two indicators: (1) pronoun use and (2) the use of words and phrases that express individualistic-collectivistic values or behaviors.

First, as culture becomes more individualistic, first-person singular pronouns (I, my, me, mine) are used more frequently while first-person plural pronouns (we, our, us, ours) are used less frequently. Prior research has indicated that first-person singular pronouns reflect individualistic tendency while first-person plural pronouns reflect collectivistic tendency (for a review, see Oyserman and Lee, 2008). Twenge et al. (2013) examined historical changes in pronoun usage in American English books between 1960 and 2008. Results showed a rise in the frequency of first-person singular pronouns and a drop in the frequency of first-person plural pronouns, suggesting an increase in individualism in the U.S. for this period^1^.

The second indicator that has been used in linguistic analysis to examine changes in individualism-collectivism is words reflecting individualistic/collectivistic values or behaviors. Twenge et al. (2012) investigated the relative frequency of words (e.g., “independent,” “individual,” “unique”) and phrases (e.g., “focus on the self,” “I am special,” “I am the best”) that reflect individualism in American English books published between 1960 and 2008. They found that both words and phrases increased for this period, suggesting an increase in individualism. Further, Greenfield (2013) indicated that along with urbanization and increased wealth, words related to adaptation to urban environments (e.g., “choose,” “unique,” “individual”) appeared more frequently in American English books published between 1800 and 2000^2^.

#### 2.1.5. Social values

Hamamura (2012) investigated temporal changes in individualistic values in the U.S. Some items showed a rise in individualism^3^. For example, the rate of people who chose “independence” as an important quality that children could be encouraged to learn at home increased between 1981 and 2006. Similarly, the percentage of respondents who chose “to obey” as most important for a child to learn to prepare him or her for life decreased between 1986 and 2008. Moreover, the rate of youths who, when asked to select a time when they felt that they were living a fulfilling life, chose “when I'm involved with something helpful for society” decreased between 1977 and 2007.

#### 2.1.6. Summary

Indices of family structure (divorce rate and household size), naming practices, words in books, and social values show that individualism has increased over time in the U.S. The incline in individualism in the U.S. has been supported by other empirical examinations such as song lyrics (DeWall et al., 2011), dictionaries (Oishi et al., 2013), and State of the Union addresses given by U.S. presidents (Oishi et al., 2013) (for reviews, see Twenge, 2015; Greenfield, 2016). Moreover, research investigating temporal changes in different but closely related concepts to individualism-collectivism, such as self-esteem and narcissism, has shown similar increases over time in the U.S. (for a review, see Twenge, 2015).

### 2.2. Factors driving american culture toward greater individualism

Why has American culture changed toward greater individualism? Researchers have offered explanations to this question. One prominent answer is that increased economic wealth promotes individualism (for a review, see Ogihara, 2017a). The economic environment has a significant effect on human psychology and behavior. Having abundant resources allows individuals more freedom to pursue their own interests, which reduces the need to depend on others. Thus, wealth affords separation from others (for reviews, see Triandis, 1995; Kraus et al., 2011, 2012). In contrast, in an environment that has few resources, accomplishing things by oneself is relatively difficult, which increases the necessity to depend on others. In this situation, rejection by other people greatly affects to one's survival and should be avoided. Thus, people who have less wealth must pay more attention to other people and the surrounding context.

In fact, the relationship between wealth and individualistic tendency at the individual level has been found in many countries, including the U.S., Japan, China, among others (for a review, see Hamamura, 2012). Further, the positive link between individualism and wealth has been found at the area level in both the U.S. (Vandello and Cohen, 1999) and Japan (Yamawaki, 2012). Additionally, this positive relationship between economic wealth and individualism has also been found at the national level (e.g., Hofstede, 1980; Diener et al., 1995; Inglehart and Baker, 2000; Kashima and Kashima, 2003).

Notably, this linkage is also supported at the temporal level in the U.S. Grossmann and Varnum (2015) examined why American culture has become more individualistic over time by investigating the relationships between five socio-ecological factors (socioeconomic status, urbanization, religion, disaster prevalence, and pathogen prevalence) and three indicators of individualism (interpersonal structure, naming practices, and word usage in books) at the temporal level. Their analyses revealed that socioeconomic status was coherently and most strongly correlated with indicators of individualism among these five socio-ecological indicators, which is consistent with the theory outlined above.

## 3. Temporal changes in individualism in Japan

As mentioned at the beginning of this paper, the amount of research investigating temporal changes in Japanese culture is relatively small compared to the accumulated literature examining temporal changes in American culture. Considering the possibility that there are cultural differences in the patterns of temporal change, it is necessary to investigate historical changes in culture other than America, which may lead to reveal how culture in general changes over time and how culture and people make each other up.

First, I will briefly summarize changes in the socioeconomic environments of Japan that are thought to affect shifts in individualism (3-1). Then, I will review a series of studies showing increases in individualism (3-2) and the persistence of **collectivism** (3-3).

KEY CONCEPT 2 **Collectivism**

Collectivism is defined as “a social pattern consisting of closely linked individuals who see themselves as parts of one or more collectives (family, co-workers, tribe, nation); are primarily motivated by the norms of, and duties imposed by, those collectives; are willing to give priority to the goals of these collectives over their own personal goals; and emphasize their connectedness to members of these collectives” (Triandis, 1995, p. 2). Notably, “[t]he core element of collectivism is the assumption that groups bind and mutually obligate individuals” (Oyserman et al., 2002, p. 5).

### 3.1. Changes in socioeconomic environments in Japan

#### 3.1.1. Economic development

**Figure 1:**
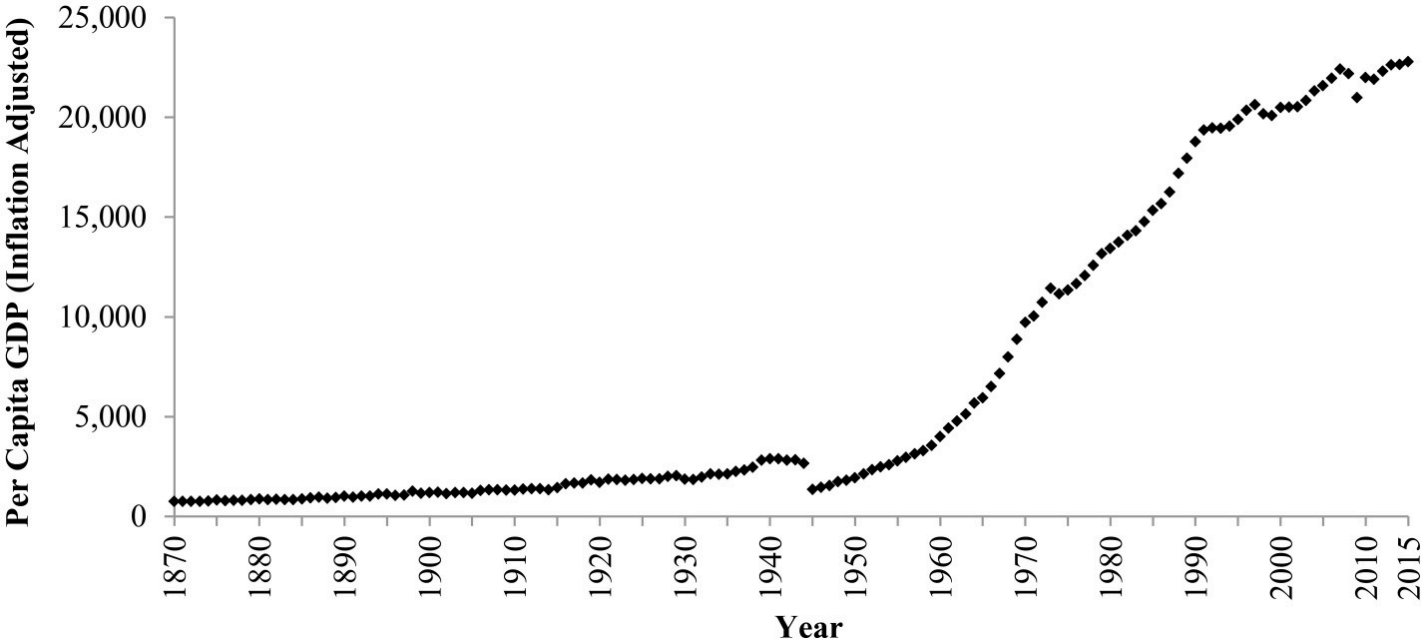
**Inflation-adjusted per capita GDP in Japan, 1870–2015**. Data between 1870 and 1989 are from the Maddison Project (2013) and data after 1990 are from The Conference Board (2016). This indicator is expressed in 1990 US dollars and purchasing power parity (PPP).

Japan has experienced dramatic and rapid economic growth. For example, per capita GDP (adjusted for inflation) steadily and remarkably increased between 1870 and 2015 (Figure 1). Considering that economic wealth promotes individualism (e.g., Inglehart and Baker, 2000; Greenfield, 2009; Grossmann and Varnum, 2015; Ogihara, 2017a), it is predicted that Japanese culture has become more individualistic for this period.

#### 3.1.2. Urbanization

**Figure 2:**
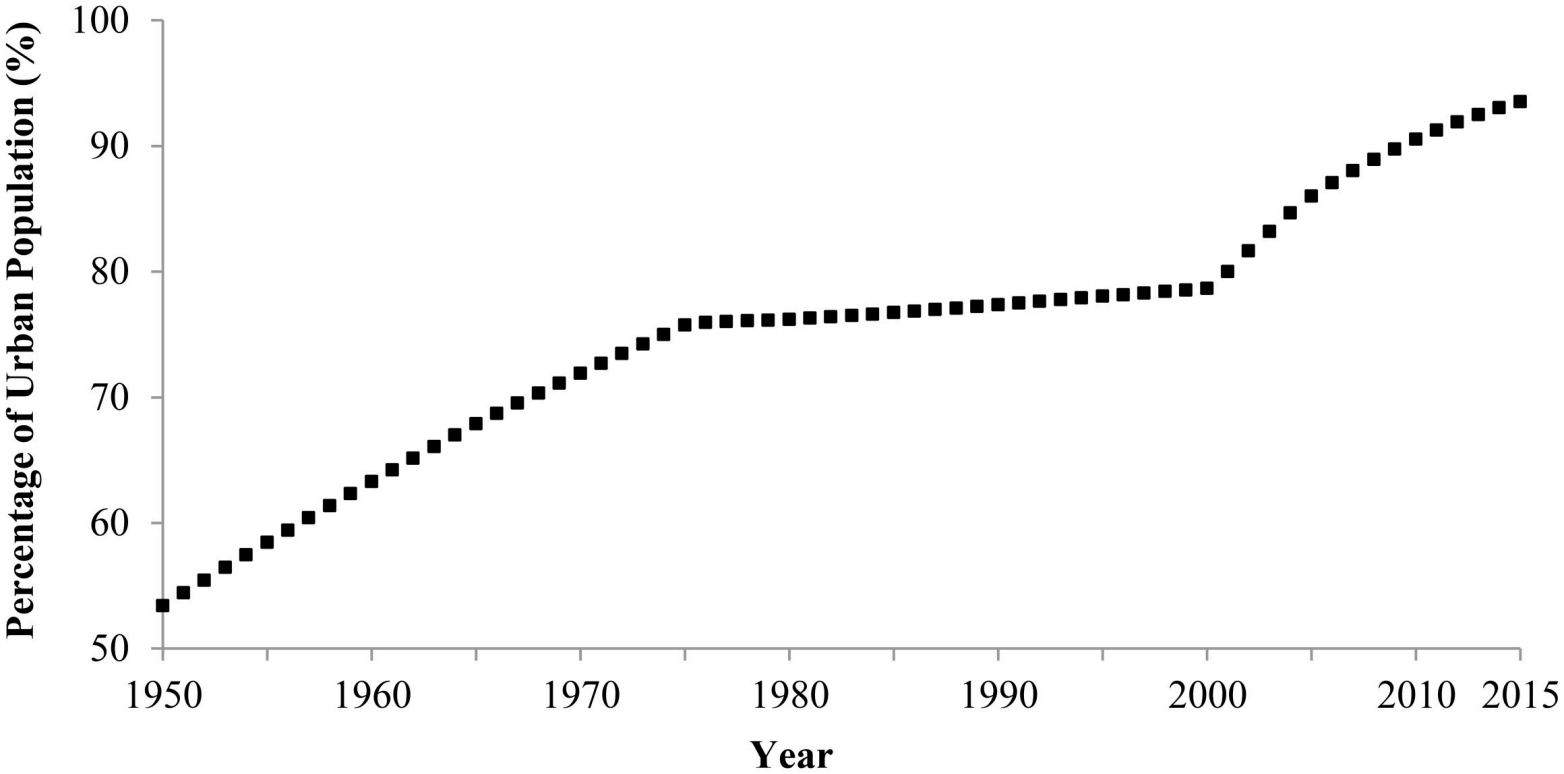
**Percentage of Japanese population living in urban areas, 1950–2015**. Data are from United Nations, Department of Economic and Social Affairs, Population Division (2014). The 2015 score is an estimation.

Japanese culture has become more urbanized. The rate of people who live in urban areas remarkably increased between 1950 and 2015 (Figure 2). In 1950, 53.4% of people lived in urban areas, but in 2015, 93.5% of people resided in urban areas. Given that urbanization encourages individualism (e.g., Greenfield, 2009; Yamagishi et al., 2012), Japan is expected to change toward greater individualism.

#### 3.1.3. Social systems

Social systems based on individualistic concepts appear to have become more prevalent in Japan. Japanese companies have abolished traditional employment systems such as the seniority system and the lifetime employment system. Indeed, the proportion of companies that determined salaries based on employee age or length of service decreased between 1996 and 2016, regardless of employee's position (i.e., management/non-management) and company size (Japan Productivity Center, 2016; Ministry of Health Labour and Welfare, 2017). In their place, they have introduced a pay-per-performance system, in which employees receive a salary that is based on their explicit individual performance rather than their age or length of service. In fact, the proportion of companies that introduced the annual salary system, which determines employee salaries based on individual short-time performance, increased between 1991 and 2014 (Japan Productivity Center, 2016; Ministry of Health Labour and Welfare, 2017). Another example can be seen in educational contexts. Schools have adopted systems that encourage students' and children's independence and uniqueness (e.g., Cave, 2001; Doi, 2004). Considering these changes in socioeconomic systems, Japan is predicted to shift in greater individualism.

### 3.2. Empirical research showing the increase in individualism

Empirical research has examined temporal changes in individualism in Japan, which basically indicates an increase in individualism over time. The findings are summarized in Table 1.

**Table 1:** **Summary of research examining temporal changes in individualism in Japan**.

	**Indicator**	**Time period**	**Main results**	**References**
Increase in individualism	Divorce rate	1950–2004 (55)	Increase in divorce rate	Hamamura, 2012
		1947–2015 (69)	Increase in divorce rate	Ogihara, Manuscript submitted
	Household size	1950–2006 (57)	Decrease in household size	Hamamura, 2012
		1953–2015 (63)	Decrease in household size	Ogihara, Manuscript submitted
	Rate of people living alone	1953–2015 (63)	Increase in rates of people living alone	Ogihara, Manuscript submitted
	Rate of nuclear family households	1964–2015 (52)	Increase in rates of nuclear family	Ogihara, Manuscript submitted
	Rate of three-generation households	1967–2015 (49)	Decrease in rates of three-generation households	Ogihara, Manuscript submitted
	Human baby names	2004–2013 (10)	Decrease in rates of common human baby names.	Ogihara et al., 2015a
		1984–2015 (32)	Decrease in rates of common human baby names	Ogihara, 2016b
	Dog names	2006–2014 (9)	Decrease in rates of common dog names	Ogihara et al., 2015b
	Words in newspaper	1875–2015 (141)	Increase in rates of headlines including individualistic words	Ogihara, 2017b
	Social values	1981–2005 (25)	Increase in importance of independence for child socialization	Hamamura, 2012
		1953–2008 (56)	Decrease in importance of family life (vs. business engagement)	
		1953–2008 (56)	Decrease in importance of following tradition	
Persistence of collectivism	Social values	1958–2008 (51)	Persistence of importance of society/nation	Hamamura, 2012
		1978–2008 (31)	Persistence of importance of social harmony	
		1981–2005 (25)	Persistence of duty of loving and respecting parents	
		1990–2005 (26)	Persistence of importance of friend	
Increase in collectivism	Social values	1963–2008 (46)	Decrease in importance of individual rights	Hamamura, 2012
		1963–2008 (46)	Increase in importance of honoring obligations	
		1977–2007 (21)	Increase in importance of effort for a successful life	
		1977–2007 (21)	Increase in importance of social contribution for fulfilling life	

*The numbers in parentheses in the time period row indicate the number of years covered by each indicator. Except for social values, they show the number of data points examined. The indicators of social values were generally collected every 5 years*.

#### 3.2.1. Divorce rates

**Figure 3:**
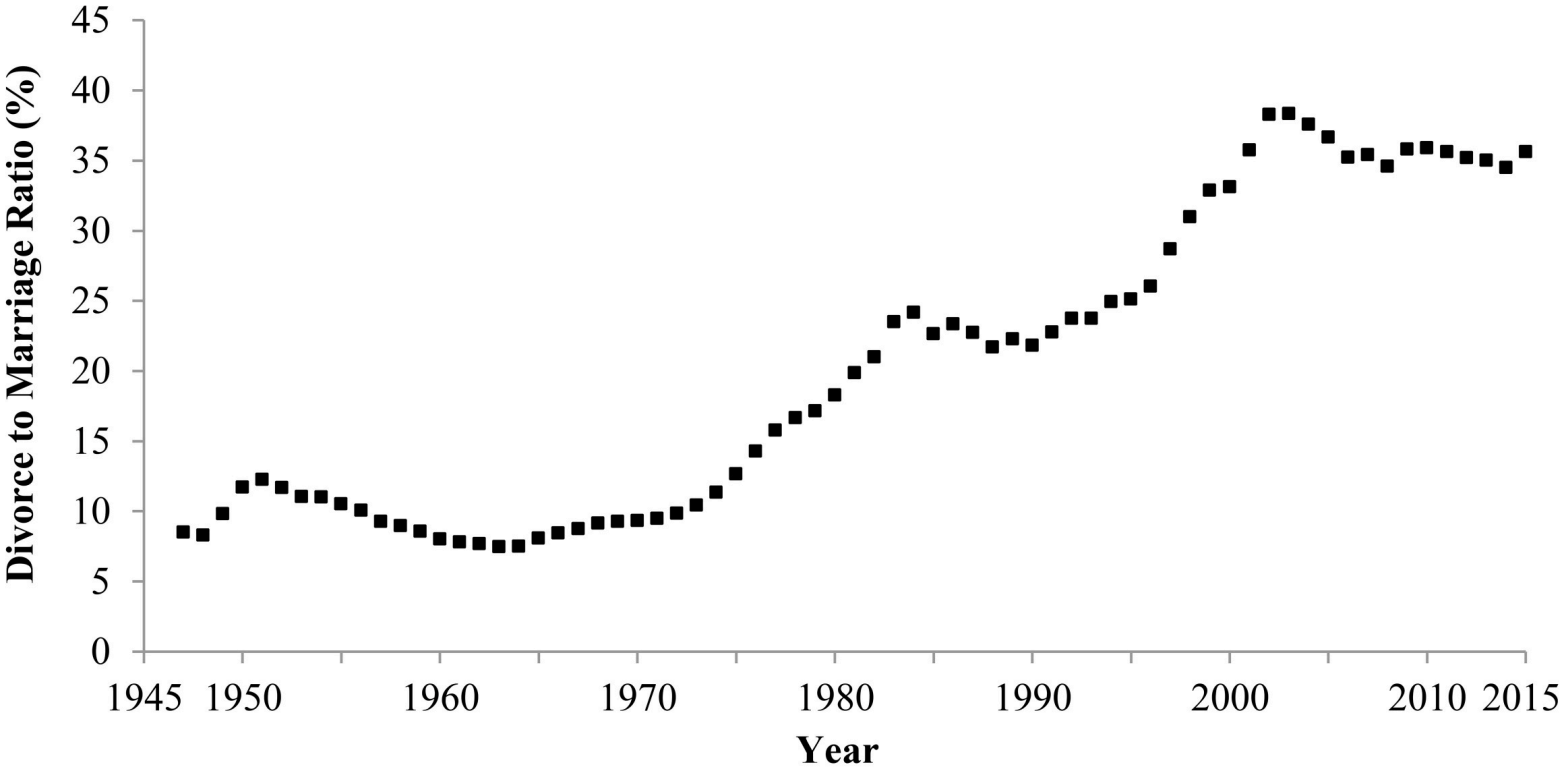
**Divorce rate in Japan, 1947–2015**. Data are from the Ministry of Health Labour and Welfare (2016a).

In Japan, the divorce rate increased between 1947 and 2015 (Figure 3). In 1947, 8.5 out of 100 couples divorced, but in 2015, 35.6 out of 100 couples experienced divorce. The correlation between year and divorce rate was strongly positive, indicating an increase in individualism (Hamamura, 2012; Ogihara, Manuscript submitted).

This change in the divorce rate was positively correlated with the increase in economic wealth (Ogihara, Manuscript submitted), which is consistent with the theory and empirical findings that wealth and individualism are positively related to each other.

#### 3.2.2. Household size

**Figure 4:**
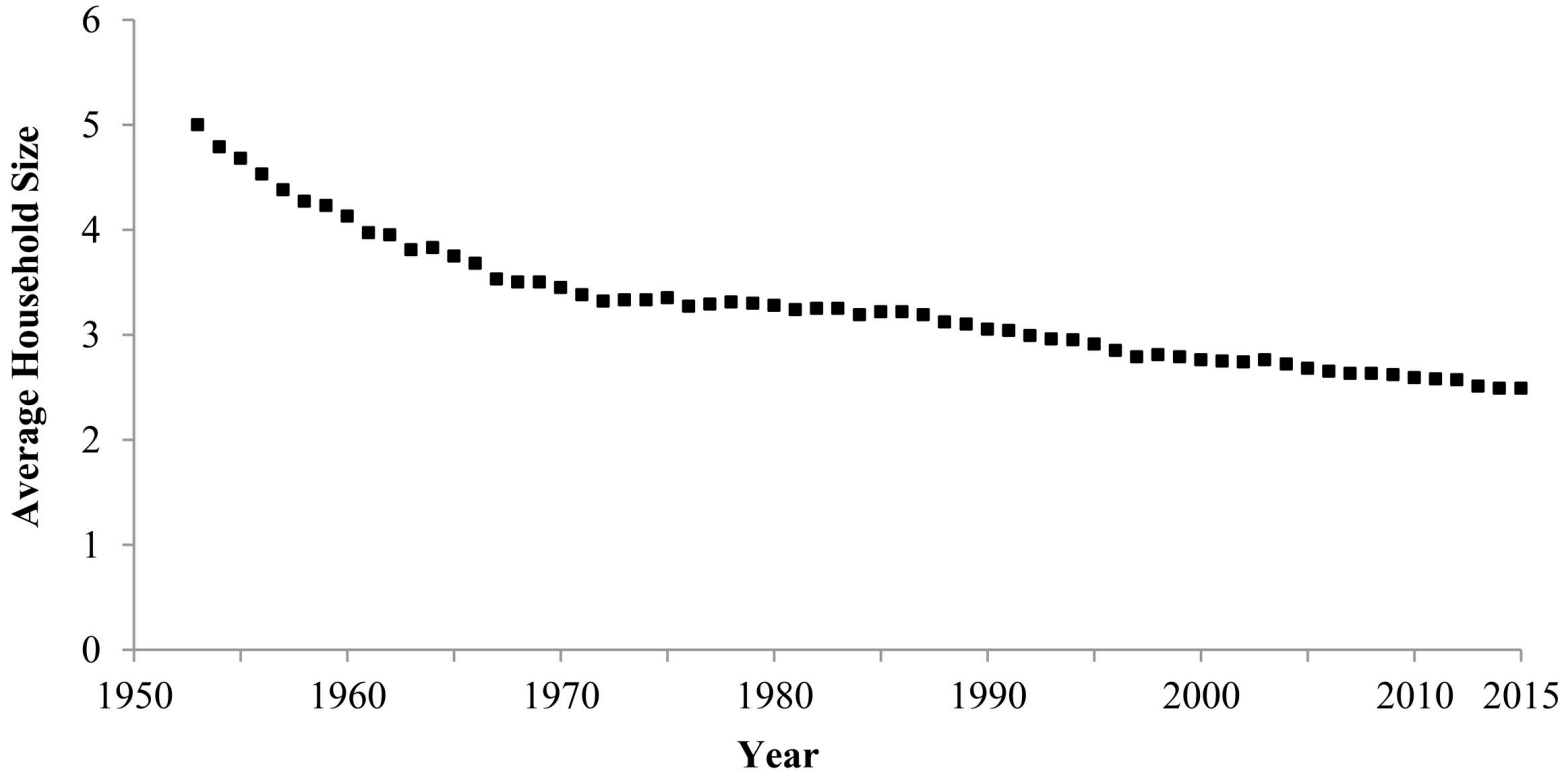
**Average household size in Japan, 1953–2015**. Data are from the Ministry of Health Labour and Welfare (2016b).

Household size became steadily smaller in Japan between 1953 and 2015 (Figure 4). In 1953, the average household consisted of five people, but in 2015, the average was 2.5 people. The correlation between year and household size was highly negative, showing a rise in individualism (Hamamura, 2012; Ogihara, Manuscript submitted).

This shift in household size was negatively correlated with the rise in wealth^4^ (Ogihara, Manuscript submitted), supporting the theory that wealth and individualism are positively associated with each other.

#### 3.2.3. Human baby names

Other than Hamamura (2012), empirical research on cultural changes in individualism in Japan was scarce. Therefore, Ogihara et al. (2015a) examined whether Japanese culture changed toward greater individualism by investigating an index other than that of family structure: baby names. They modeled this study on Twenge et al. (2010)'s approach of examining temporal changes in the U.S. by investigating the rates of unique baby names.

In Japan, unique names have attracted much attention not only in academia but also in the society at large (Ogihara, 2015). Anthropologists and sociologists had insisted that unique names increased, but there was no empirical data to support these claims. Thus, to clarify temporal changes in cultural practices, an empirical investigation of whether people give more unique names was necessary.

It is difficult to collect sufficient data on names in Japan unlike in the U.S. where the government systematically collects the names of almost all newborns and publishes rankings of common names (Twenge et al., 2010; Social Security Administration, 2016). Thus, they analyzed data collected and published by two private companies (an educational service company and an insurance company) in Japan between 2004 and 2013.

They found that the rates of names using common pronunciations decreased for both male and female newborns for the 10 years. In contrast, the rates of common Chinese characters increased over the same time period. These results were consistent among two independent datasets from two private companies. Moreover, it was also revealed that variation in the pronunciation of the same combinations of Chinese characters increased, whereas variation in the written forms of the same readings decreased over the same period, showing that parents express uniqueness in pronunciation rather than in writing. These results suggest that parents use common Chinese characters, but give them uncommon pronunciations to create a unique name. One way that parents use to create unique names is to provide an English pronunciation for a Chinese character. For instance, a common Chinese character 海 (meaning marine or sea)” is usually pronounced as “Kai” or “Umi” in Japan, but parents read this “Marin” after the English pronunciation “marine” (for other ways of giving unique names in Japan, see Ogihara, 2015). Such unique twists in assigning pronunciation to names suggest that Japanese increasingly seek uniqueness, indicating an increase in individualism in Japan.

This research is important because, other than Hamamura (2012), the amount of research examining temporal changes in individualism in Japan was limited and this research indicates the rise in individualism with an index other than that of family structure (Hamamura, 2012), which strengthens the validity of the finding indicating individualism has increased in Japan. Furthermore, this study provided empirical evidence of the increase in uniqueness and individualism in naming practices that was expected by anthropologists and sociologists.

Moreover, Ogihara (2016b) examined temporal changes in baby names in the years preceding the 2000s by investigating names published in birth announcements in municipality newsletters from 1980s. Many municipalities publish newsletters to share important information both with their members and with those living outside the municipality. Such newsletters include the names of those who have recently been born, married, or died in the municipality. Using the internet, as many newsletters as possible that met certain criteria were collected.

Analysis found the same cultural trends for this time period as was found in the research investigating trends in the 2000s (Ogihara et al., 2015a). Therefore, this suggests the phenomenon of giving more unique names to babies was not specific to 2000s, but was present going back at least as far as the 1980s. This indicates that the change toward greater individualism in Japanese culture dates back at least 30 years.

#### 3.2.4. Dog names

Is the search for uniqueness found only when people give names to human babies? If Japanese culture has changed toward greater individualism, people would give unique names to other targets as well. Thus, Ogihara et al. (2015b) investigated whether dog names have also become more unique.

They conducted a similar analysis on dog names as was used to analyze trends in human baby names. They collected data of dog names from a pet insurance company in Japan and computed the rates of common dog names for each year between 2006 and 2014 as a preliminary analysis.

They found that the rates of common dog names decreased over this period, which was consistent with the results in human baby names. These results also suggest that Japanese culture has sought more uniqueness and become more individualistic.

#### 3.2.5. Words in newspapers

The indicators above (divorce rate, household size, naming practices for human babies and dogs; see Table 1) have shown that individualism has risen since World War II. However, it was unclear whether such changes also occurred before World War II. It is important to reveal how culture changes over time for a longer period (e.g., Grossmann and Varnum, 2015). Therefore, Ogihara (2017b) investigated historical trends in individualism in Japan between 1875 and 2015 by examining temporal changes in the relative frequencies of words reflecting individualistic values or behaviors in one Japanese national newspaper^5^ as a preliminary analysis.

He calculated the relative frequencies of headlines which included individualistic words (e.g., “individual,” “uniqueness,” “chose”) in the national newspaper that had the most readers and the longest database in Japan (*The Yomiuri Shimbun*), between 1875 and 2015. Results showed that the percentages of individualistic words increased for this time, suggesting that Japanese culture became more individualistic over the 140 year period.

#### 3.2.6. Social values

So far, I have reviewed the indicators of individualistic behaviors (i.e., separating from a spouse, living separately from family members, giving unique names to human babies and dogs, using individualistic words). Not only indicators of behaviors, but also indicators of values show temporal shifts toward greater individualism in Japan (Hamamura, 2012). For example, the percentage of respondents who chose “independence” as an important quality that children could be encouraged to learn at home increased between 1981 and 2005 in Japan. Moreover, the rate of people who chose family life over important business engagement decreased between 1953 and 2008 for both males and females. These items were validated as indicators of individualistic-collectivistic values by checking correlations with other indices of individualism at the national level and the regional level in Japan.

#### 3.2.7. Summary

A series of studies investigating family structure (i.e., divorce rate and family size), human baby names, dog names, words in newspapers and social values consistently suggest that Japanese culture has become more individualistic over time.

These patterns are consistent with those found not only in American culture, but also in other East Asian cultures. Recently, research has suggested an increase in individualism in China (for reviews, see Sun and Ryder, 2016; Ogihara, 2017a). The divorce rate steadily increased between 1978 and 2015 and household sized shrank between 1953 and 2015 (Ogihara, Manuscript submitted). Further, analyses of temporal changes in words reflecting individualistic values/behaviors have shown similar trends (Hamamura and Xu, 2015; Zeng and Greenfield, 2015). Moreover, South Korea also seems to have experienced cultural change toward greater individualism over the past two decades (Park et al., 2016). Taken together, this suggests that East Asian cultures have become more individualistic over time.

### 3.3. Empirical research showing the persistence of collectivism

I have reviewed a series of studies indicating the rise in individualism in Japan. However, did all aspects in Japanese culture become more individualistic? The findings above have shown that at least some aspects of Japanese culture have changed toward greater individualism over time, but this does not necessarily mean that all aspects of Japanese culture have shifted toward greater individualism.

Indeed, Hamamura (2012) reported that collectivistic values persist, or have even become more prevalent over time in Japan. For instance, the rate of people who agreed that one must always love and respect one's parents was fairly stable between 1981 and 2005. Similarly, the percentage of people who regarded friends as important was almost unchanged between 1990 and 2005. Additionally, the percentage of people who selected “respect individual rights” when they were asked to choose important moral principles decreased between 1963 and 2008.

These results are consistent with the finding that cultural change in social values is moderated by cultural heritage (Inglehart and Baker, 2000). It is important to understand that cultures are influenced simultaneously by many socio-ecological factors other than economic growth. Although the economic environment is one of the major factors, it is not the only one. There are other important socio-ecological factors, which may affect some aspects more strongly (for reviews, see Oishi and Graham, 2010; Oishi, 2014; Ogihara, 2017a).

This coexistence of individualism and traditional collectivism was reported not only in Japan, but also in China (Zeng and Greenfield, 2015).

## 4. Ramifications of the coexistence of rising individualism and persisting collectivism

Cultural shifts toward greater individualism in historically individualistic cultures and collectivistic cultures seem to have different implications. Japan may be transitioning from a collectivistic culture to an individualistic culture. Thus, there is a trend toward greater individualism, despite the simultaneous persistence of collectivism. People have difficulty in adapting to this ambiguous context, including conflicts, and/or contradictions at various levels. This coexistence of rising individualism and traditional collectivism seems to have a negative impact on people in Japan, especially in the aspect of interpersonal relationships.

In fact, people with a high personal achievement orientation (one of the individualistic tendencies; e.g., Oyserman et al., 2002; Taras et al., 2014) feel lower Subjective Well-Being (SWB) in Japan (Ogihara and Uchida, 2014). This negative relationship was mediated by having fewer close friends. That is, people with high individualistic tendency have fewer numbers of close friends, which was in turn related to lower SWB. In contrast, in the U.S., although the number of close friends was positively related to SWB, there was no significant relationship between personal achievement orientation and the number of close friends, or between personal achievement orientation and SWB. Similarly, a high need for uniqueness was negatively correlated with income, current life satisfaction, anticipated life satisfaction, and satisfaction with personal relationships in Japan (especially in areas with low social mobility; Takemura, 2014).

Consistent with this finding, Japanese people recognize that individualism may injure close interpersonal relationships (Ogihara et al., 2014b). As a whole, they regard individualism as ambivalent. On one hand, Japanese view individualism as positive because they think individualism gives them independence and freedom. In collectivistic societies, relatively strong norms or shared beliefs sometimes prevent people from pursuing their own goals and/or preferences (e.g., Gelfand et al., 2011). In this context, individualism can give individuals independence and freedom from such constraints. On the other hand, Japanese regard individualism as negative because they believe that individualism contaminates close interpersonal relationships. These two aspects cancel each other out, resulting in an overall ambivalent attitude toward individualism. In contrast, American people regard individualism as significantly positive (Ogihara et al., 2014a). They value it highly for the freedom and independence it provides.

## 5. Conclusion

In this paper, I have reviewed empirical studies investigating temporal changes in individualism and their ramifications in Japan. I would like to highlight three conclusions that can be drawn from these studies.

First, recent studies have shown that Japanese culture has become more individualistic. This trend is consistent with trends found in both American and other East Asian cultures (e.g., China, South Korea).

Second, it is not the case that all aspects of Japanese culture have changed toward greater individualism. Some social values have not become more individualistic. This is also found in China.

Third, the coexistence of individualism and traditional collectivism is related to undesirable interpersonal relationships and SWB in contemporary Japan. This is possibly a problem that a traditionally and historically collectivistic culture may experience when it becomes more individualistic.

## 6. Future directions

Finally, I raise three future directions that are important to reveal how cultures change over time and how people and culture make each other up.

### 6.1. Uncovering the factors explaining the difference between changing toward greater individualism and persisting in collectivism

This paper reviewed how Japanese culture has changed over time by introducing studies showing both an increase in individualism and the endurance of collectivism. However, it is not clear what explains the difference between them. Uncovering this difference is crucial to understand mechanisms of cultural change over time.

To answer this question, more research into various aspects of temporal changes in individualism, such as type of indicator (e.g., behavior/value), domain (e.g., independence/uniqueness/personal achievement) and level (e.g., explicit/implicit, individual/collective) may be effective. While there are studies investigating cultural changes in Japan, the amount of research on this topic is still scarce. Comparing the direction, speed, and extent of change within different aspects is necessary. Moreover, revealing the reasons for such plausible differences is required. This could reveal which aspects of culture are changeable/flexible and which are fixed/inflexible. In addition, it would be desirable to replicate cultural variables in the lab for experimental manipulation to examine under what conditions change occurs, and which variables affect aspects of this change (e.g., direction, rate) (for a review of useful approaches, see Kashima, 2014).

### 6.2. Investigating temporal changes in other psychologies and behaviors

In this article, I focused on reviewing studies about temporal changes in individualism in Japan. Yet, how have other psychological tendencies changed over time? Although individualism-collectivism is one of the most important and frequently researched concepts, of course there are also other important psychological concepts, whose temporal changes should be investigated.

For example, studies have investigated temporal changes in self-esteem, which is also one of the most important and often researched concepts in psychology (e.g., Baumeister et al., 2003; Ogihara, 2016a). In the U.S., it increased over time (e.g., Twenge and Campbell, 2001; Gentile et al., 2010). In contrast, in Japan, it decreased (Oshio et al., 2014; Ogihara, 2016c; Ogihara et al., 2016)^6^.

This phenomenon in Japan is intriguing and thought-provoking because, at a first glance, it seems paradoxical. Specifically, considering that Japanese culture has become more individualistic, it would be expected that self-esteem has increased, but it has actually decreased (for a more detailed discussion, see Ogihara, 2016c; Ogihara et al., 2016). Why has self-esteem decreased rather than increasing in Japan? Why have these cultural differences in temporal changes in self-esteem emerged? By answering these questions, we could better grasp how cultures change over time.

### 6.3. Revealing the negative ramifications of the coexistence of individualism and collectivism

I mentioned the coexistence of individualism and collectivism may have negative results in Japan. However, this process has been unclear. This is especially important in cultures that are traditionally collectivistic, but are increasingly individualistic (e.g., East Asian cultures), because we can expect them to suffer from these conflicts or problems in the present and the near future. Many studies have suggested that interpersonal relationships is one major factor predicting positive mental/physical health, so the deterioration of interpersonal relationships is one of the key factors of causing mental and physical illness. Although there may be other factors, maladaptation to the relatively new social environments caused by cultural change in Japan may be one important reason. Thus, revealing how and why interpersonal relationships are destroyed would contribute to solving and preventing social issues.

## Author contributions

The author confirms being the sole contributor of this work and approved it for publication.

## Funding

YO was supported by a Grant-in-Aid for Japan Society for the Promotion of Science fellows (JSPS KAKENHI Grant Number 26-5799) and the Kyoto University Foundation. This work was supported by special funding for the promotion of internationalization of research activities by the Japanese Group Dynamics Association.

### Conflict of interest statement

The author declares that the research was conducted in the absence of any commercial or financial relationships that could be construed as a potential conflict of interest.

## Footnotes

^1^A similar change was found in other languages such as French, German, Spanish, Italian, Russian, Chinese, and Hebrew between 1949 and 2008 (Yu et al., 2016).

^2^These patterns were found not only in American English books, but also in British English books between 1800 and 2000 (Greenfield, 2013).

^3^It should be noted that other items indicated different patterns. Some items showed the persistence of individualism. For instance, the importance of friends was stable between 1990 and 2006 (but this may be due to the fact that the rates were close to 100% for all four time points). Other items indicated increased collectivism. For example, the rate of respondents who agreed that one must always love and respect one's parents increased between 1981 and 1999.

^4^These trends were also found when other similar indicators were used instead of household size. Specifically, the percentage of people living alone increased both for the total population and for the elderly aged over 65, regardless of sex. Furthermore, the rate of households with three generations living together decreased and the rate of nuclear family households increased over time. These shifts were correlated with household size and the economic changes in the predicted directions (for details, see Ogihara, Manuscript submitted).

^5^Previous research used Google Ngram Viewer, a huge corpus of published books in several languages, to investigate historical changes in individualism explained above (2.1.4. Words in books). Following the methodology of previous studies (e.g., Twenge et al., 2012, 2013; Greenfield, 2013; Yu et al., 2016), it was desirable to use this corpus for exploring cultural changes in Japan, but there is no Japanese version of this corpus at present time. Thus, Ogihara (2017b) used newspaper databases as an alternative.

^6^The decline in self-esteem was also found in China between 1996 and 2009 (Xin et al., 2012; Liu and Xin, 2015).
